# Astrocyte response to motor neuron injury promotes structural synaptic plasticity via STAT3-regulated TSP-1 expression

**DOI:** 10.1038/ncomms5294

**Published:** 2014-07-11

**Authors:** Giulia E. Tyzack, Sergey Sitnikov, Daniel Barson, Kerala L. Adams-Carr, Nike K. Lau, Jessica C. Kwok, Chao Zhao, Robin J. M. Franklin, Ragnhildur T. Karadottir, James W. Fawcett, András Lakatos

**Affiliations:** 1 John van Geest Centre for Brain Repair, Department of Clinical Neurosciences, University of Cambridge, E.D. Adrian Building, Forvie Site, Robinson Way, Cambridge CB2 0PY, UK; 2 Department of Veterinary Medicine & Wellcome Trust-MRC Stem Cell Institute, University of Cambridge, Madingley Road, Cambridge CB3 0ES, UK; 3 These authors contributed equally to this work

## Abstract

The role of remote astrocyte (AC) reaction to central or peripheral axonal insult is not clearly understood. Here we use a transgenic approach to compare the direct influence of normal with diminished AC reactivity on neuronal integrity and synapse recovery following extracranial facial nerve transection in mice. Our model allows straightforward interpretations of AC–neuron signalling by reducing confounding effects imposed by inflammatory cells. We show direct evidence that perineuronal reactive ACs play a major role in maintaining neuronal circuitry following distant axotomy. We reveal a novel function of astrocytic signal transducer and activator of transcription-3 (STAT3). STAT3 regulates perineuronal astrocytic process formation and re-expression of a synaptogenic molecule, thrombospondin-1 (TSP-1), apart from supporting neuronal integrity. We demonstrate that, through this new pathway, TSP-1 is responsible for the remote AC-mediated recovery of excitatory synapses onto axotomized motor neurons in adult mice. These data provide new targets for neuroprotective therapies via optimizing AC-driven plasticity.

## Abstract (web summary)

Perineuronal astrocyte reactivity is implicated in functional recovery following nerve injury but exactly how this happens is unclear. Tyzack *et al*. show that perineuronal astrocytes facilitate the recovery of synaptic inputs to damaged neurons via STAT3-dependent upregulation of the astrocytic protein TSP-1.

## Graphical abstract

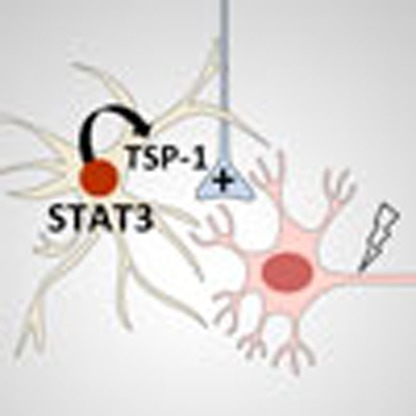

Plasticity of synaptic input around cell bodies of neurons occurs rapidly following injury to their central or peripheral axon projections. Proximal to the lesion, this adaptive remodelling mechanism may both switch functioning neurons into the circuitry^1^,^2^ and enhance neuronal viability^3^. A critical but unresolved issue is the role of perineuronal AC reactivity in this process. Could remotely activated grey matter glial cells orchestrate dynamic structural changes in neuronal connections? Synapse elimination and rearrangement around the neuronal soma and dendrites occur concomitantly with glial cell activation^4^,^5^,^6^. Similar to microglia, perineuronal ACs are known to respond to distant insults by extending hypertrophic processes around synapses. However, the extent to which this type of reactive transformation represents a detrimental or a protective response for neuronal function and integrity in the adult central nervous system (CNS) has never been directly addressed. This is highly pertinent, as targeting reactive AC–neuronal interactions proximal to damage is a plausible alternative approach for facilitating neuronal survival and repair. This is due to the relative lack of negative influence from other invading cell types^6^,^7^. Thus, understanding the precise mechanisms and consequences of remote AC activation may hold the key to optimizing the efficiency of functional recovery.

Most reports on the astrocytic behaviour have derived from studies on ACs residing in the vicinity of a lesion, but often led to conflicting views. Although reactive ACs have been shown to express a number of molecules inhibitory for plasticity^8^,^9^, recent studies argue that they can also be beneficial for repair. Within traumatic spinal cord lesions, signal transducer and activator of transcription-3 (STAT3) signalling was found to regulate AC activation, which was also shown to be necessary for wound healing and functional recovery^10^,^11^,^12^. The exact mechanism underlying synapse recovery in the vicinity of these lesions is unknown. AC-derived thrombospondin-1/2 (TSP-1/2) attracted interest as they have been found to increase excitatory synapse density in the developing CNS^13^,^14^. Recent studies using experimental ischaemic CNS damage reported that thrombospondin-1/2 can be re-expressed locally at the lesion site^15^,^16^, but their regulation in reactive ACs also remains unresolved.

The complexity of these lesions may cloud direct interpretations of default AC behaviour, and the above findings may not be relevant to situations when ACs are remotely activated in a less inflammatory environment^17^,^18^,^19^. The influx of other cells around the lesion and the release of cytokines that may reach distant areas by diffusion and via the cerebrospinal fluid can influence both AC response and synaptic recovery.

We directly addressed the effect of remote AC reactivity on neuronal integrity and recovery of their synaptic input while reducing potential confounding factors. We used unilateral extracranial facial nerve transection where the AC response is distant and predominantly triggered by neuronal insults in a less inflammatory environment. By selectively impeding AC activation in an established transgenic system^12^, we could reliably examine its effect on neuronal function. We show that remotely activated grey matter ACs directly promote structural synaptic plasticity and support network integrity. We also provide the underlying mechanism, showing that STAT3 activation upregulates astrocytic TSP-1 re-expression and release, which is required to facilitate the recovery of synaptic input onto surviving motor neurons after their distant axonal insult.

## Results

### STAT3 induces AC process formation after axotomy

**Figure 1:**
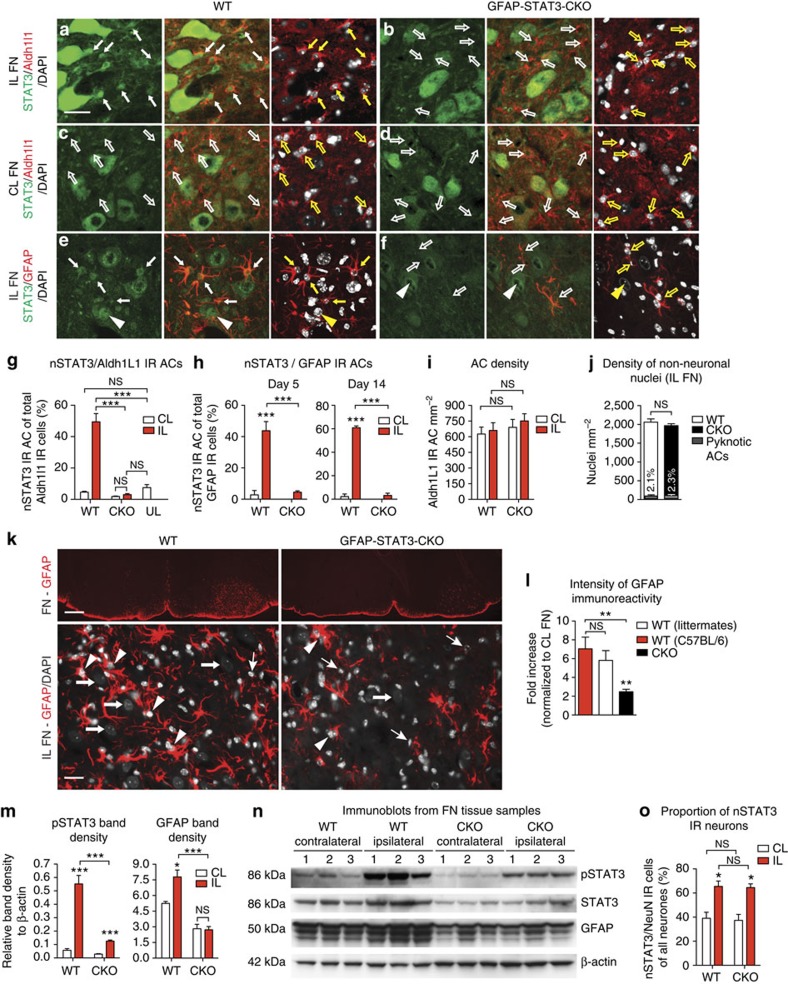
STAT3 activation is necessary for reactive transformation of perineuronal ACs after remote injury. (**a**–**f**,**k**) Grey matter AC response in the facial nucleus (FN) 14 days post-facial nerve transection. (**a**–**d**) Nuclear localization of STAT3 in Aldh1L1 IR ACs (fluorescent microscopy), (**e**,**f**) in GFAP IR reactive ACs (thin arrows; confocal images) and in motor neurons (arrowheads) in the IL FN of WT mice (**a**,**e**) and its relative absence (empty arrows) in the CL side in WT mice (**c**) and in CKO mice (**b**,**d**,**f**). (**g**) Proportion of Aldh1L1 IR ACs and (**h**) GFAP IR ACs displaying nSTAT3 labelling at day 5 or 14 post axotomy in WT mice (*n*=3) and in CKO mice (*n*=5) (**g**, ***P<0.001, one-way analysis of variance (ANOVA); **h**, *n*=3, ***P<0.001, one-way ANOVA *P*<0.0001). (**i**) AC density is expressed as mean of Aldh1L1 IR cell count (mm^−2^), which is comparable in WT and CKO groups and remains unchanged after axotomy (*n*=4, ^NS^*P*=0.645, one-way ANOVA). (**j**) Numbers of intact non-neuronal nuclei (*n*=3, ^NS^*P*=0.39, *t*-test ) and the proportion of pyknotic ACs in both groups (*n*=3, ^NS^*P*=0.634, *t*-test ). (**k**) GFAP immunoreactivity in the axotomized and CL FN in WT mice and in CKO mice. GFAP IR ACs (arrowheads) surround axotomized neurons (full arrows) and display marginal pyknosis (thin arrows) in the two groups. (**l**) Mean intensity (optical density) values for IL GFAP immunoreactivity normalized to the CL side within the same section (WT *n*=3, CKO *n*=6, ^NS^*P*=0.49, *t*-test; **P<0.01, one-way ANOVA *P*=0.0013). (**m**) Means of band densities for pSTAT3 and GFAP immunoblots (**n**, Supplementary Fig. 1) of IL and CL FN tissue samples, demonstrating overall STAT3 phosphorylation induced by axotomy in WT mice and to a lesser extent in CKO mice (*n*=3, ***P<0.001, *P<0.05, one-way ANOVA). (**o**) Mean percentages of neurons displaying nSTAT3 labeling. Scale bar, 30 μm for **a**–**f**; 250 μm and 20 μm for **k**; *n*=mice per group. Data represent mean±s.e.m.

To explore whether STAT3 signalling plays a key role in the AC response to remote axonal injury, we used glial fibrillary acidic protein (*GFAP*)*-Cre/STAT3-loxP* conditional knockout (CKO) mice in comparison with wild-type (WT) controls. Astrocytic STAT3 activation was verified by nuclear translocation of immunoreactivity (IR) in the facial nuclei (FN) 14 days after facial axotomy (Fig. 1a–f). To identify all ACs, tissue sections were immunostained for ALDH1L1, a pan-astrocytic marker that is less dependent on STAT3 activity^20^ (Fig. 1a–d). In WT mice (*n*=3) the proportion of ACs with STAT3 activation was significantly higher at 49.44±5.26% compared with the contralateral (CL) side (4.6±0.54%) or for unlesioned controls (7.51±1.9%; *n*=3, *P*<0.001; Fig. 1a,c,g). In CKO mice (*n*=5), STAT3 activation was effectively abolished in ACs (Fig. 1b,d,g).

We then addressed whether STAT3 activation is associated with characteristic features of reactive AC transformation, such as GFAP expression. First, we examined whether the STAT3-activated population overlaps with GFAP-expressing ACs (Fig. 1e,f). In WT mice, 43.77±5.84% of GFAP-positive cells displayed nuclear STAT3 (nSTAT3) IR at day 5, which rose to 60.9±1.54% at day 14 when compared with the CL side at each time point (*n*=3, *P*<0.001; Fig. 1e,h). In CKO mice, this response was negligible or absent (Fig. 1f,h). Quantifications of AC reaction assessed by intensity (optical density) measurements of GFAP immunoreactivity showed that intensity values were equally (*P*=0.49) increased 7-fold and 5.8-fold in the affected FN of WT C57BL/6 mice and WT littermate controls compared to the CL side (*n*=3; Fig. 1k,l). By contrast, in the CKO group, GFAP density values only rose 2.5-fold (*n*=6, *P*<0.01). AC death was not the cause of decreased GFAP immunoreactivity, as pyknosis in this population was equally marginal in the two groups (*P*=0.634; Fig. 1j,k). Furthermore, AC numbers were also comparable as assessed by labelling for ALDH1L1 (*n*=4, *P*>0.05 for all; Fig. 1i).

We also tested whether STAT3 phosphorylation, another aspect of STAT3 activation, is associated with increased GFAP expression by immunoblotting whole FN tissue samples. In the ipsilateral (IL) FN of WT mice, the density of pSTAT3 IR bands was increased by 9.6-fold (*P*=0.001) when compared with the CL side. While this response was much lower in CKO mice, overall STAT3 phosphorylation was not entirely abolished (*n*=3, Fig. 1m,n). This mirrors the diminished extent of nSTAT3 IR observed in CKO mice, and it also indicates that other cells, such as neurons can efficiently activate STAT3 after axotomy (Fig. 1e,f,o). We also found a positive correlation (*R*=0.9, *P*=0.004) between the density of pSTAT3 and GFAP IR bands in the axotomized FN. The latter was greater (*P*<0.001) by 2.9-fold in WT mice than in CKO mice (*n*=3; Fig. 1m,n).

**Figure 2:**
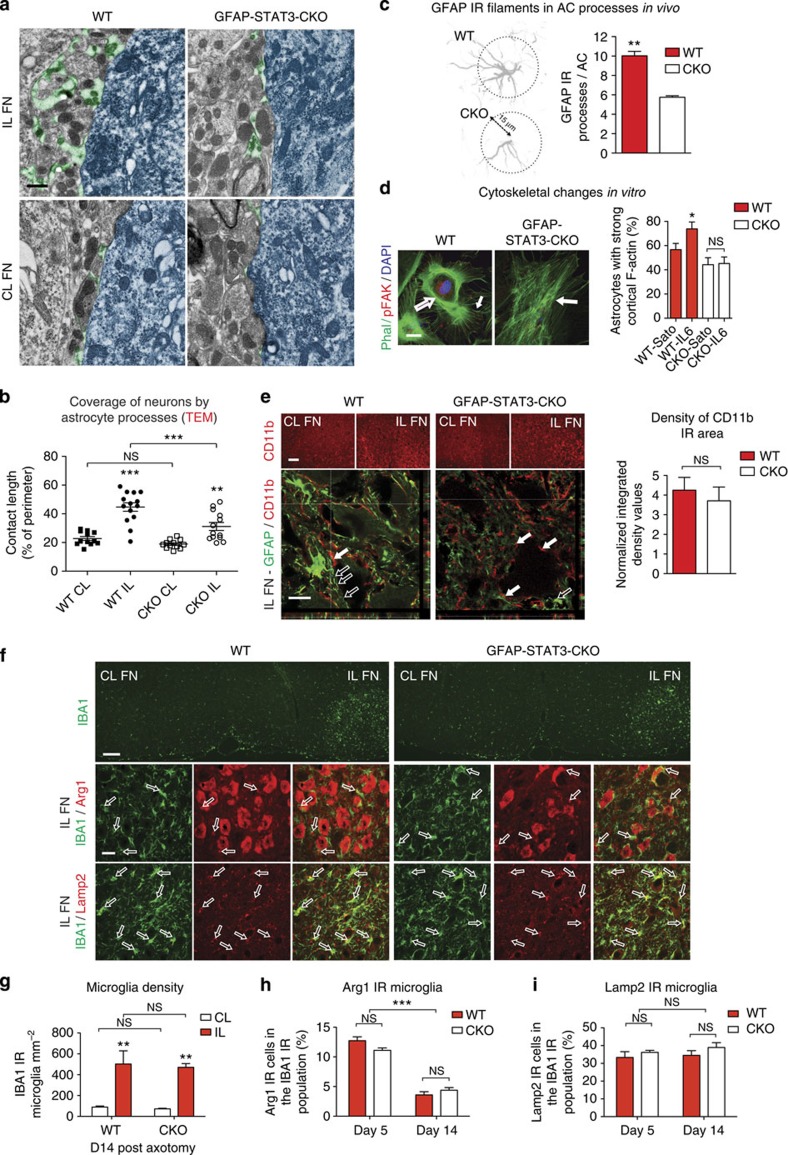
STAT3 mediates reactive AC process formation. (**a**,**b**) Differential increase in perineuronal AC process availability 14 days post facial nerve transection in WT and CKO mice. (**a**) Electron micrographs of AC endfeet (green) along the neuronal membrane (blue) in the IL and CL FN. (**b**) Ultrastructural analysis: percentage of neuronal perimeter contacted by AC endfeet (CL *n*=12, IL *n*=14 cells, ***P<0.001, **P<0.01, one-way analysis of variance (ANOVA) *P*<0.0001). (**c**) Mean number of AC processes that are GFAP IR 15 μm beyond the soma (*n*=3 mice, 63–90 cells in total, **P=0.009, *t*-test). (**d**) *In vitro* examples of activated ACs displaying cortical F-actin fibres (phalloidin, empty arrow) with connections to radial filaments (small arrow) and phosphorylated focal adhesion kinase (pFAK) 24 h after IL-6 treatment. Non-activated ACs retain their flat morphology with mostly parallel filaments (full arrow). Mean numbers of WT or CKO ACs that retained their activated phenotype 24 h after activation by IL-6 or in serum-free medium (Sato) (*n*=3 experiments per replicate cultures, *P=0.043, *t*-test). (**e**) CD11b immunolabelling in the CL and IL FN in WT and CKO mice. Lower panel represents confocal images of the axotomized FN. In WT mice, CD11b IR structures (full arrows) appear to intermingle with processes that are strongly positive for GFAP (empty arrows). Mean density values for CD11b IR areas normalized to the CL FN in WT and CKO mice at day 14 post axotomy. (**f**) Illustrations of IBA1 IR-activated microglia in IL FN (green) and of its subtypes co-labelled (arrows) for markers, Arg1 or Lamp2 (red) in WT and CKO mice at day 14 post axotomy. (**g**) Means of cell densities for IBA1 IR-activated microglia at day 14. (**h**,**i**) Means of percentages of Arg1/IBA1 (**h**) or Lamp2/IBA1 (**i**) double-labelled microglia/macrophages over the total number of IBA1-positive cells in the IL FN (day 5 *n*=4 and day 14 *n*=3, ***P<0.001, **P<0.01, one-way ANOVA). Scale bar, 0.25 μm for **a**; 20 μm for **d**; 100 and 20 μm for **e**; 200 and 25 μm for **f**. Data represent mean±s.e.m.

By electron microscopy we then determined whether STAT3 activation can influence AC process availability, a sensitive measure of AC reaction. The abundance of AC processes was confirmed by ultrastructural studies in which the proportion of neuronal membrane contacted by AC endfeet was quantified^21^. We detected a significantly greater (*P*<0.001) neuronal coverage by WT AC processes in the axotomized FN compared with that seen for the CKO mice (WT IL: 44.7±2.94%, CKO IL: 31.2±2.79%, *n*=14 cells; Fig. 2a,b). The astrocytic coverage in the non-affected FN was comparable between the groups (Fig. 2a,b). Having confirmed that the abundance of perineuronal processes depends on astrocytic STAT3 activity, we examined whether it also correlates with changes in cytoskeletal filament content, such as GFAP, in individual ACs. The number of AC processes that were positively labelled for GFAP was quantified 15 μm away from their soma (63–90 cells per group analysed, *n*=3; Fig. 2c). We found a 1.74-fold increase in process numbers in the WT group where ACs displayed an average of 10.02±0.46 processes (*P*=0.009) compared with 5.75±0.15 processes seen for the CKO mice. To assess whether the increase in the number of GFAP IR processes correspond with STAT3-induced dynamic cytoskeletal changes, we quantified the number of cells displaying cortical F-actin assembly with connections to radial filaments in cultured ACs activated by interleukin-6 (IL-6). At 24 h, significantly more WT ACs retained their reorganized cytoskeleton (*P*=0.043), while CKO cells did not respond and showed flat morphology with parallel filaments (*n*=3 experiments per replicate cultures; Fig. 2d). We conclude that STAT3 activation transforms grey matter ACs into a reactive phenotype, thereby increasing their perineuronal presence following axotomy.

Finally, we asked whether there are differences in the relative availability of reactive ACs to microglia to define the degree of specific astrocytic influence on neurons. Thus, we assessed the microglial response in WT and CKO mice after axotomy. We quantified both the density of CD11b-positive areas and the presence of activated proinflammatory microglia/macrophages labelled for IBA1/Arg1 or for IBA1/Lamp2, respectively. We found comparable density values for CD11b immunoreactivity in the axotomized FN in the two groups at day 14 (*n*=3, *P*=0.299; Fig. 2e). Although there was a more than 5.6-fold increase (*P*=0.001) in density of total IBA1-positive activated microglia after axotomy, their numbers were comparable (*P*=0.82) in the presence of normal and diminished AC reactivity (*n*=4; Fig. 2f,g). Moreover, the proportions of Arg1-labelled cells among IBA1-positive microglia was equal between the two groups as early as day 5 (*n*=3, *P*=0.098) and also at day 14 when cell densities significantly declined (*P*<0.001) by more than 3.5-fold (*n*=4, *P*=0.307; Fig. 2f,h). We did not find differences in the fraction of IBA1/Lamp2-positive microglia/macrophages up to day 14 (*n*=3, *P*=0.218; Fig. 2f,i). These results indicate that microglia/macrophage numbers were not affected significantly by the reduction in astrocytic STAT3 activation.

### ACs support neuronal integrity via STAT3 signalling

**Figure 3:**
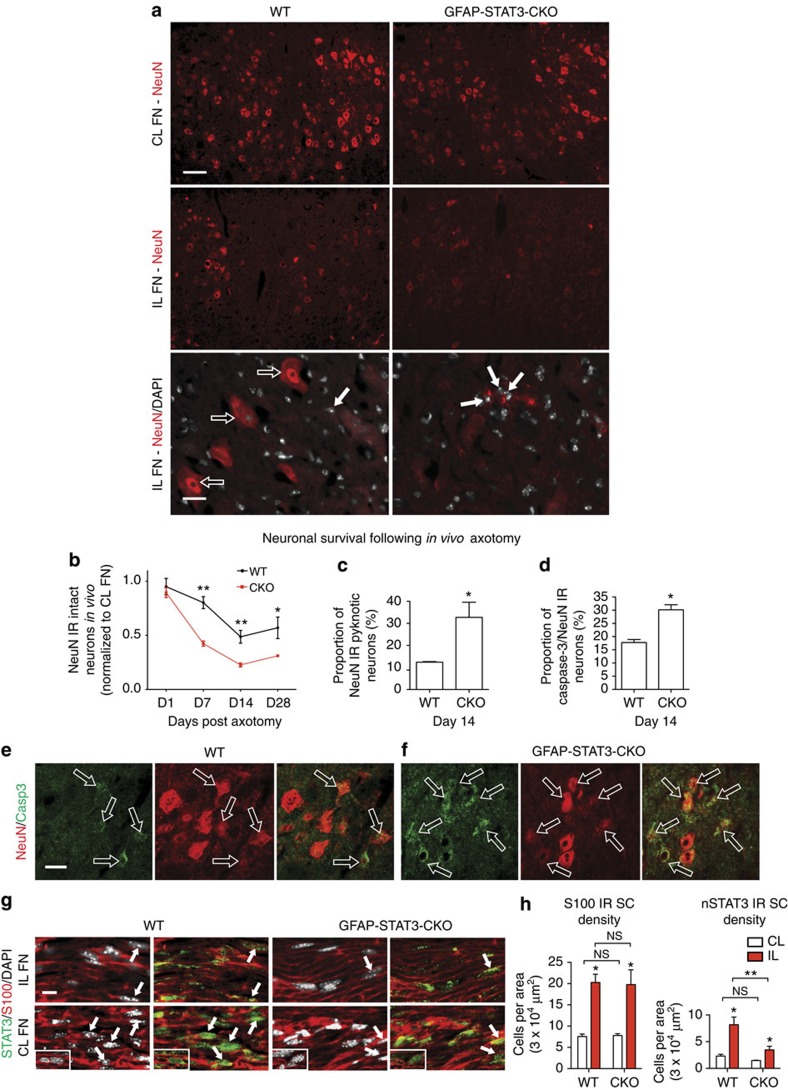
Neuronal integrity is maintained in areas of full AC activation *in vivo*. (**a**) Fluorescent microscopic images of NeuN IR neurons in the CL and IL FN in brain sections from WT and CKO mice 14 days after facial nerve transection. DAPI-stained pyknotic neuronal nuclei are present in the IL FN (full arrows) among intact neurons (empty arrows). (**b**) Relative number of intact and strong NeuN IR neurons at days 1, 7, 14 and 28 post axotomy (*n*=3, 3; 4, 4; 7, 7; 4, 3 mice for WT, CKO groups, two-way analysis of variance (ANOVA) *P*<0.0001, *F*(_1/24_)=23.72 for the genotype effect, **P=0.0026, **P=0.0029, *P=0.044, respectively, *t*-test for pairs). (**c**) Mean percentages represent the proportion of neurons undergoing pyknosis (*n*=3 mice, *P=0.041, *t*-test) and (**d**) the proportion of NeuN IR neurons that are also positive for activated caspase-3 labelling (*n*=3 mice, *P=0.025, *t*-test). (**e**,**f**) Examples of activated caspase-3/NeuN IR neurons in the IL FN of WT and CKO mice. (**g**) Axotomized and also CL facial nerves sampled close to the stylomastoid foramen 1 mm away from the nerve stump. Images show nuclear localization of STAT3 (nSTAT3, arrows) in S100-positive SCs in both WT and CKO mice. Insets represent confocal microscopic images. (**h**) Means of density values (cells per area) for the total number of S100-positive cells and for nSTAT3/S100 double-labelled cells in the intact CL and axotomized nerves (*n*=3 mice, *P<0.05, **P=0.002, one-way ANOVA *P*=0.0016). Scale bar, 100 μm and 25 μm for **a**; 30 μm for **e**,**f**; 10 μm for **g** and for insets. Data represent mean±s.e.m.

We addressed whether ACs maintain neuronal viability through STAT3 signalling. First, we compared neuronal integrity in the FN of WT mice and CKO mice with diminished AC reactivity and STAT3 activation (Fig. 3a). Viable, non-atrophic neurons were defined by strong NeuN IR and intact nuclei at days 1, 7, 14 (Fig. 3a,b) and 28 post-facial nerve transection. From day 7, the preservation of neuronal integrity was nearly twofold greater (*P*<0.0001) in the vicinity of activated WT ACs (Fig. 3a,b). In CKO mice, the proportion of pyknotic nuclei with weak NeuN immunoreactivity was 2.63-times higher (*P*=0.041) than in WT mice at day 14 (*n*=7; Fig. 3a,c). Neuronal death was also confirmed by activated caspase-3/NeuN co-labelling. 30.14±1.93% of neurons displayed activated caspase-3 immunoreactivity in the IL FN of CKO mice, which was 1.7-fold greater than that seen for WT mice (*n*=3, *P*=0.025; Fig. 3d–f).

We also addressed whether Schwann cells (SCs) could be affected by GFAP-Cre-dependent reduction in STAT3 activation in CKO mice, which could potentially contribute to neuronal death by the loss in their trophic influence at the nerve stump. We found a reduction in the density of nSTAT3-positive and S100 IR myelinating or non-myelinating SCs in axotomized nerves sampled 1 mm proximal to the stump (*P*=0.002). However, the overall density of SCs raised to equal values in WT and CKO mice following axotomy (*n*=3, *P*=0.857; Fig. 3g,h).

**Figure 4:**
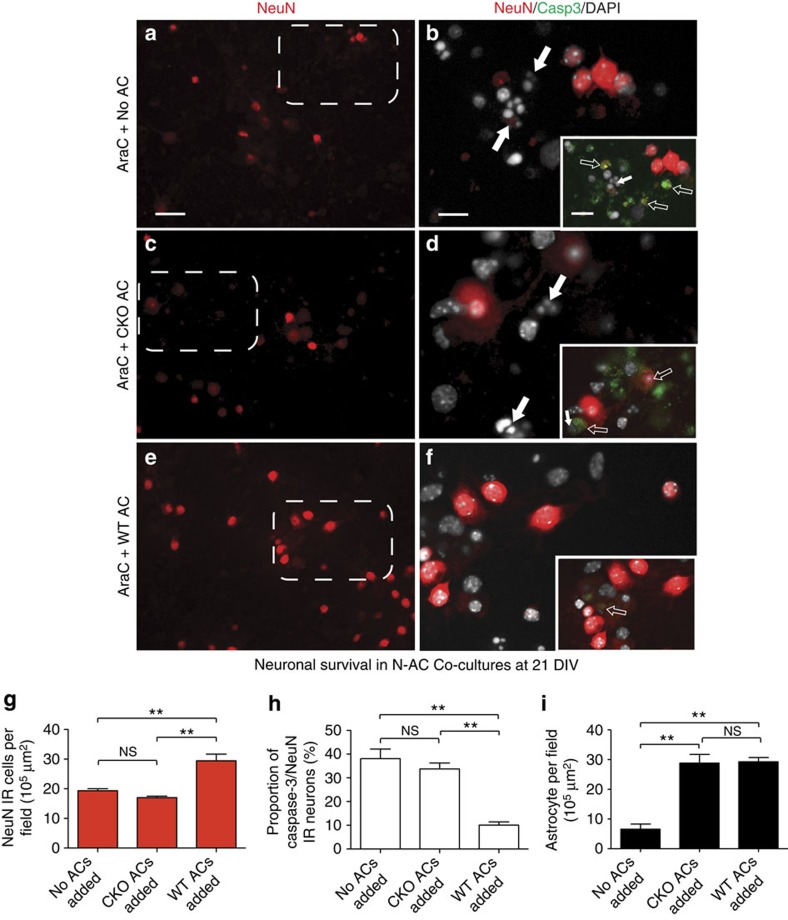
Astrocytic STAT3 supports neuronal integrity independently of other cell types *in vitro*. (**a**–**f**) Fluorescent images of NeuN/DAPI co-labelled cells in purified neuronal cultures in the near absence of ACs (**a**,**b**) and in the presence of CKO ACs (**c**,**d**) or WT ACs (**e**,**f**). Examples of pyknotic cells (full arrows, **b**,**d**). Insets illustrate the same areas with activated caspase-3/NeuN co-labelling. Activated caspase-3-positive neurons with reduced NeuN immunoreactivity (empty arrows) are prevalent in the ‘no added AC’ (**b**) and CKO AC (**d**) conditions. Some activated caspase-3-positive cells are already pyknotic (small arrows) and/or disintegrated into debri (empty arrows). (**g**) Mean number of surviving NeuN IR neurons per experiment at 21 DIV (*n*=3 cultures per 9 fields each, **P<0.01, one-way analysis of variance (ANOVA) *P*=0.0016). (**h**) Mean percentage values of NeuN IR neurons that are also co-labelled for activated caspase-3. (**i**) The numbers of WT and CKO ACs surviving at 21 DIV were comparable. Only a small number of WT ACs remained in the ‘no added ACs’ group after AraC treatment (for **h** and **i**: *n*=3 cultures per 9 fields each, **P<0.01, one-way ANOVA *P*=0.001). Scale bar, 30 μm for **a**,**c**,**e**; 10 μm for **b**,**d**,**f** and for insets. Data represent mean±s.e.m.

We then sought to determine *in vitro* whether ACs via STAT3 can exert a direct neuroprotective effect independently of SCs. We set up a purified AC–neuron co-culture system in which ACs were initially ablated by AraC and then replaced either by WT, CKO or no ACs for comparison (*n*=3). Cortical neurons derived from neonatal mice are known to be vulnerable in culture, especially without the presence of astrocytic factors, providing a simple survival assay^22^. Viable neurons were defined by the criteria used in our *in vivo* experiments (Fig. 4a–f). Consistent with our *in vivo* findings, WT ACs were nearly 1.73 times more efficient (*P*<0.01) than CKO ACs in supporting neuronal integrity (Fig. 4g). This was also mirrored by a significantly reduced proportion of activated caspase-3-positive neurons in the presence of WT ACs (10.1±1.4%; *n*=3, *P*=0.001) when compared with the CKO group (33.8±2.49%) or to cultures with no added ACs (38.1±4.04%; *n*=4, *P*<0.01; Fig. 4h), while the number of re-plated ACs in the WT and CKO groups was equal (Fig. 4i). Together, these results suggest that ACs exert an independent STAT3-mediated protective effect.

### AC reaction is associated with synaptic recovery

**Figure 5:**
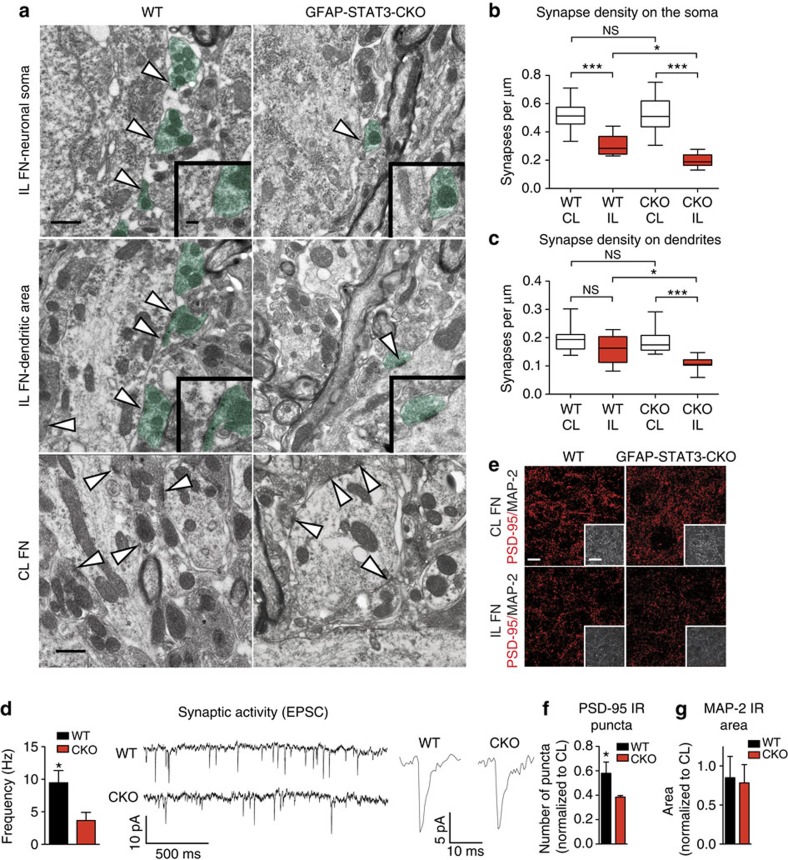
STAT3-mediated AC response corresponds with greater synaptic density and input onto neurons. (**a**) Electron micrographs of axosomatic and axodendritic synapses within a 50 μm radius from the soma in the IL and CL FN 14 days post axotomy. WT mice, in contrast to CKO mice, have more presynaptic terminals (green) opposing the postsynaptic density (arrowheads). Insets demonstrate postsynaptic densities. (**b**) Number of synaptic contacts (μm^−1^) along the surface of the soma (WT *n*=12 cells, CKO *n*=17 cells) and (**c**) within the dendritic area (WT *n*=13 dendrites, CKO *n*=12 dendrites, * *P*<0.05, ***P<0.001, one-way analysis of variance *P*<0.0001). (**d**) Frequency of excitatory postsynaptic currents from whole-cell recordings in the IL FN 12–14 days post axotomy (WT *n*=5, CKO *n*=6, *P=0.036, *t*-test), and representative trace recordings with single EPSCs. (**e**) Confocal images of immunostained acute brainstem slices of axotomized postnatal (P21–23) mice processed after electrophysiological studies. Examples demonstrating PSD-95 IR synapses (red) and MAP-2 IR dendrites (insets, white) in the CL and IL FN at 12–14 days post axotomy. (**f**) Means of number of PSD-95 IR puncta (*n*=3 mice, *P=0.041, *t*-test) and (**g**) MAP-2 IR areas (*n*=3 mice, ^NS^*P*=0.176, *t*-test) normalized by CL values in the same section. Scale bar, 0.5 μm for **a**; 0.1 μm for insets; 10 μm for **e** and 25 μm for insets. Data represent mean±s.e.m.

We also asked whether the AC reaction enhances the maintenance or recovery of synaptic input on neurons to help restore function. To address this question, initially we compared synapse densities in WT and CKO mice at day 14 post axotomy. This time point correlates with accelerated synapse formation, which follows the initial phase of synapse stripping^6^. At the ultrastructural level axosomatic synapse density was defined by the number of synapses along a 1 μm length of the neuronal membrane (Fig. 5a). The values were equal in the CL sides in the two groups, suggesting normal developmental synaptogenesis in the CKO group. However, synapse density in the injured side was 1.54-fold higher in WT mice (*n*=12 cells) compared with CKO mice (*n*=17 cells, *P*<0.05; Fig. 5b). The density of axodendritic synapses reached the values of the CL side in WT mice (*n*=13 dendrites), whereas this was still reduced by 1.76-fold in the CKO group (*n*=12 dendrites, *P*<0.001; Fig. 5c). These results suggest that AC reactivity promotes structural synaptic rearrangements.

To assess whether these observed changes represent functional synapses, we measured frequencies of excitatory postsynaptic currents in surviving motor neurons 10–14 days after axotomy. Whole-cell patch clamp recording was performed on acute brainstem slice preparations taken from axotomized postnatal mice (P20–P24). In CKO mice, there was a clear reduction in synaptic activity (3.67±1.25 Hz, *n*=6, *P*=0.036) in surviving neurons when compared with that seen in WT mice (9.47±1.87 Hz, *n*=5; Fig. 5d). This suggests a more abundant excitatory synaptic input post axotomy in WT animals over CKO mice, which also correlates with a 1.45-fold greater density of PSD-95-labelled postsynaptic puncta in WT mice (*P*=0.036), while the density of dendrites has not changed (Fig. 5e–g).

**Figure 6:**
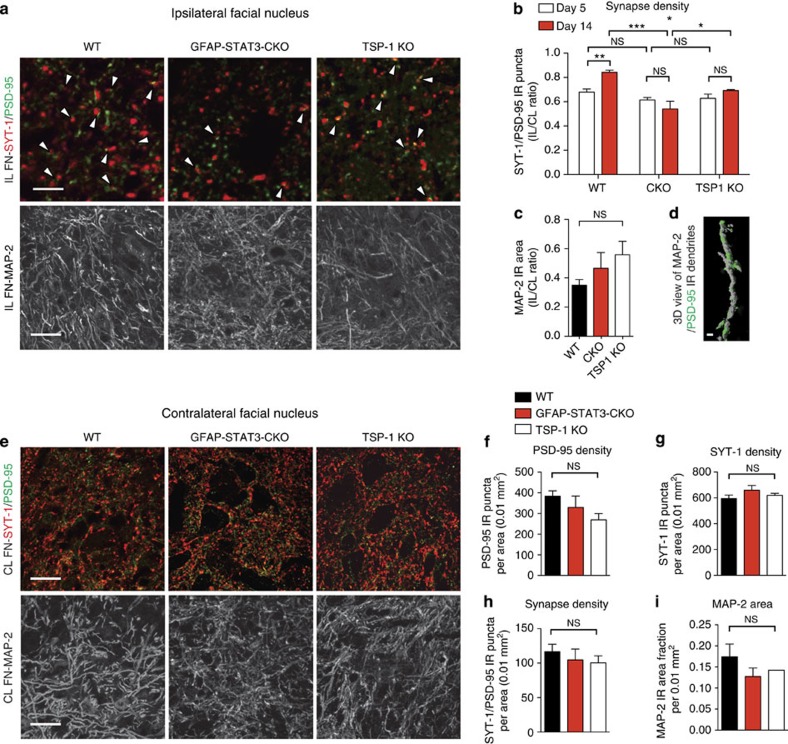
Recovery of excitatory synaptic density is associated with astrocytic STAT3 activation and is TSP-1 dependent. (**a**) Confocal images of synapses (arrowheads) demonstrated by colocalization of synaptotagmin-1 (SYT-1) and PSD-95 IR puncta and the MAP-2 IR dendritic tree in the IL FN in WT, CKO and TSP-1 KO mice 14 days post axotomy. (**b**) Means of ratios of synapses in the IL FN relative to the CL FN at day 5 (*n*=3 mice, one-way analysis of variance (ANOVA) *P*=0.299) and at day 14 post axotomy (WT *n*=6, CKO and TSP-1 KO *n*=4 mice, ***P<0.001, *P<0.05, one-way ANOVA *P*=0.0002 and *t*-test between day 5 and day 14 values: **P=0.001). (**c**) Means of ratios of MAP-2 IR areas (*n*=4 mice, ^NS^*P*=0.272, one-way ANOVA). (**d**) Three-dimensional reconstruction of confocal images showing a MAP-2 IR dendrite (grey) with areas of PSD-95 immunoreactivity at shaft and spinal synapses (green) in the WT IL FN. (**e**) Images showing synapses illustrated by SYT-1 and PSD-95 immunoreactivity and MAP-2 labeling (white) in the CL FN in adult WT, CKO and TSP-1 KO mice. Quantification of (**f**) PSD-95, (**g**) SYT-1, (**h**) SYT-1/PSD-95 IR puncta and (**i**) MAP-2 area fraction in the CL FN in the three groups (*n*=4, ^NS^*P*>0.05 for all, one-way ANOVA). Data represent the mean of number of synaptic puncta or MAP-2 IR area per 0.01 mm^2^ field. Scale bar, 10 μm and 25 μm for **a**; 1 μm for **d**; 25 μm for **e**. Data represent mean±s.e.m.

Next, we addressed whether the greater excitatory input in WT mice represents synapse recovery rather than maintenance of connections in adult animals. We identified synapses by synaptotagmin-1/PSD-95 co-labelling during synapse stripping (day 5) and the re-organization phase (day 14) in both IL (Fig. 6a) and CL FN (Fig. 6e). Synapse densities were defined by the number of IR puncta per 0.01 mm^2^ area, and were normalized to the CL measurements to demonstrate the efficacy of synapse recovery. Initially synapse densities declined equally (*P*=0.125) to values of 0.68±0.03 and 0.61±0.02 for WT and CKO mice (*n*=3), respectively. However, significant recovery was only observed in WT mice to a ratio of 0.84±0.02 (*n*=6, *P*=0.001; Fig. 6b). In contrast, in areas of STAT3-deficient AC populations, this ratio remained as low as 0.54±0.05 (*n*=4), 30% lower when compared with that seen in the WT mice (Fig. 6b). Despite greater neuronal degeneration in the CKO mice, the IL/CL ratios of MAP-2-positive dendritic areas (Fig. 6a) did not differ significantly in the two groups (*n*=4; Fig. 6c). This may suggest dendritic compensatory mechanisms^23^ in surviving neurons in CKO mice; however, synapse recovery was still abolished. Our results indicate that surviving neurons in the CKO mice receive significantly less excitatory input per cell than their WT counterparts.

### TSP-1 is required for synapse recovery following axotomy

**Figure 7:**
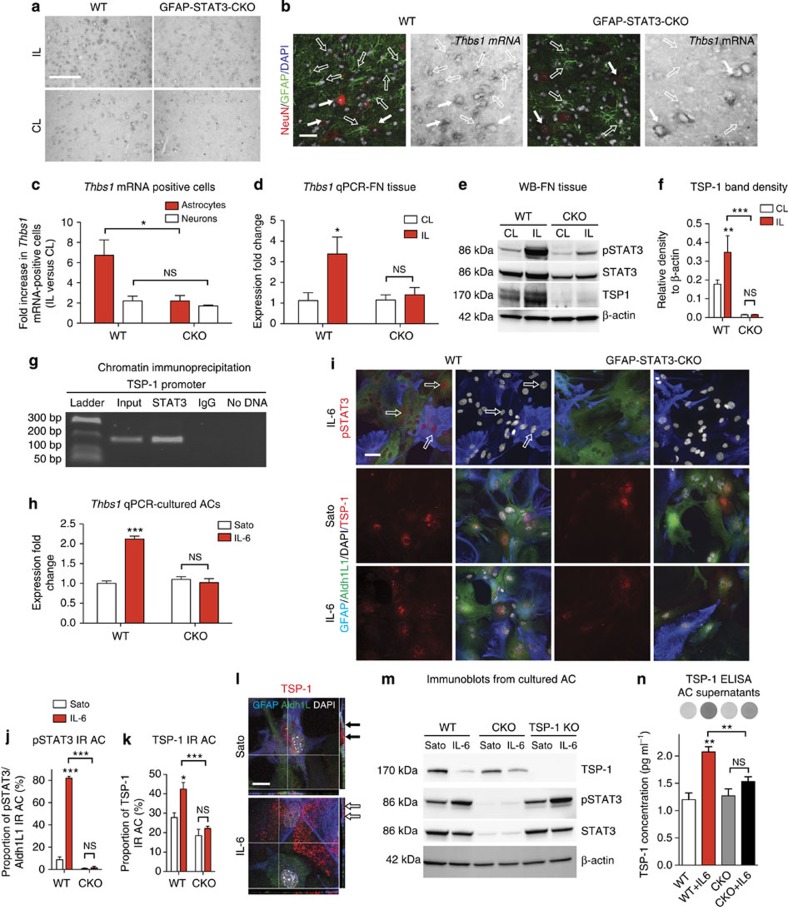
STAT3 activation directly regulates TSP-1 expression in ACs. (**a**,**b**) *In situ* hybridization for *Thbs1* in the FN 7 days post axotomy in WT and CKO mice. (**b**) Triple-labelling shows co-localization of *Thbs1* mRNA with GFAP (empty arrows) or NeuN immunoreactivity (arrows). (**c**) Quantification of ACs and neurons expressing *Thbs1* mRNA (*n*=3 mice, *P=0.039, ^NS^*P*=0.407, *t*-test). (**d**) *Thbs1* mRNA measured by qPCR in IL and CL FN tissue samples in WT and CKO mice at day 14 (*n*=3, *P<0.05, one-way analysis of variance (ANOVA) *P*=0.035). (**e**) Immunoblots of samples taken from FN tissues at day 14, representing TSP-1 content against pSTAT3, STAT3 and β-actin immunoreactivity. (**f**) Mean density values for TSP-1 immunoblots of IL and CL FN (*n*=3, **P<0.01, ***P=0.0002, one-way ANOVA). (**g**) ChIP demonstrates specific STAT3 binding to the TSP-1 promoter by showing PCR product enrichment in the STAT3 pull-down sample. Positive control: sonicated/non-precipitated input sample. Negative controls: normal rabbit immunoglobulin G and ‘No DNA’ samples. (**h**) *Thbs1* mRNA levels measured by qPCR in purified WT (*n*=3) and CKO AC cultures (*n*=6) with or without IL-6 treatment (***P<0.001, one-way ANOVA *P*<0.001). (**i**) Aldh1L1/GFAP/DAPI- and pSTAT3 or TSP-1 quadruple labelling in WT and CKO ACs activated by IL-6 or in serum-free conditions (Sato). Proportion of GFAP/Aldh1L1 IR ACs displaying (**j**) pSTAT3 (empty arrows) or (**k**) TSP-1 immunoreactivity in Sato and IL-6-activated conditions (*n*=3 experiments per replicate cultures, ***P<0.001, *P<0.05, one-way ANOVA *P*=0.0012). (**l**) Confocal images illustrating intracellular or extracellular distribution of TSP-1 immunoreactivity in non-activated or IL-6-treated WT ACs, respectively. (**m**) TSP-1, pSTAT3 and STAT3 immunoblots for WT, CKO and TSP-1 KO ACs treated with Sato or IL-6. (**n**) ELISA: mean concentrations of TSP-1 (pg ml^−1^) released into supernatants by WT and CKO ACs after treatment with Sato or IL-6. Dots represent chemiluminescence (*n*=3 experiments per replicate samples, **P<0.01, one-way ANOVA *P*=0.002). Scale bar, 200 μm for **a**; 50 μm for **b**; 40 μm for **i**; 20 μm for **l**. Data represent mean±s.e.m. Supplementary Figs 2–4.

We then asked what factors are directly responsible for the observed synaptic changes mediated by reactive ACs. TSP-1, an AC-derived molecule responsible for excitatory synaptogenesis during development, was recently shown to be re-expressed locally in CNS injuries^16^,^24^. Thus, we hypothesized that TSP-1 plays a role in the plasticity of synaptic input on axotomized motor neurons in adult mice. To explore this possibility, we compared synaptic densities in the FN of TSP-1 KO mice with those found in WT and CKO mice at days 5 and 14 (Fig. 6a,b). Successful TSP-1 protein depletion in ACs derived from TSP-1 KO mice was verified by immunoblots (Fig. 7m). Synapse recovery was significantly impaired in TSP-1 KO mice, but to a smaller degree than that found in the CKO group (see above). Between days 5 and 14, the normalized synapse density values had only returned to 0.74±0.03 from 0.69±0.01 (*n*=4, *P*<0.05; Fig. 6b). The IL/CL ratio of MAP-2 IR dendritic areas was also comparable with the values seen in all other groups (Fig. 6c). In addition, synapse density and MAP-2 area fraction values measured in the CL FN of all groups were equal, which reflects no differences in developmental excitatory synaptogenesis or dendritic areas between WT and CKO or TSP-1 mice (Fig. 6e–i). In summary, our findings provide evidence that TSP-1 plays an important role in adult plasticity following axonal insults.

### Astrocytic STAT3 induces TSP-1 expression and release

We asked whether TSP-1 could be responsible for reactive AC-driven synaptic recovery via STAT3 signalling. TSP-1 (*Thbs1*) messenger RNA (mRNA) expression was examined in the FN of WT and CKO mice at day 14 post axotomy. By *in situ* hybridization, we detected a 6.73-fold increase in the number of *Thbs1* mRNA/GFAP-expressing ACs (Fig. 7a–c) in the WT mice when compared with the CL side (*n*=3, *P*=0.039). This increase was reduced to 2.21-fold in reactive ACs with impaired STAT3 activation in the CKO mice. Motor neurons also displayed *Thbs1* mRNA expression, however, their numbers were only marginally increased by 1.71-fold on the axotomized side and was equal in the two groups (*n*=3; *P*=0.407; Fig. 7b,c). The number of *Thbs1* mRNA-positive cells excluding ACs and neurons were negligible.

To confirm the link between astrocytic STAT3 activation and *Thbs1* expression, we also quantified mRNA levels by quantitative PCR (qPCR) from FN tissue samples. In WT mice, *Thbs1* mRNA levels were ~3-fold greater in the axotomized FN when compared with the non-affected FN or to the FN of CKO mice (*n*=3, *P*=0.035; Fig. 7d). This response to axotomy was also reflected by increased TSP-1 protein expression indicated by a 1.9-fold higher band density value in immunoblots from IL FN tissue samples of WT mice (*n*=3, *P*=0.002; Fig. 7e,f). Selective reduction of AC reactivity in CKO mice resulted in no response in *Thbs1* mRNA (*P*=0.589) and almost completely abolished TSP-1 protein expression when compared with that seen in WT mice (*P*=0.001).

We then asked whether STAT3 directly regulates *Thbs1* expression in ACs. We tested this by a series of molecular studies using purified ACs cultured with or without the STAT3 activator IL-6. First, chromatin immunoprecipitation (ChIP) assay was performed using DNA samples from cultured ACs activated by IL-6. The TSP-1 promoter PCR product was enriched in the STAT3 pull-down sample, indicating that STAT3 specifically binds to the promoter sequence (Fig. 7g). To confirm that STAT3 binding also initiates transcription, we then measured *Thbs1* mRNA levels from these samples by qPCR. We found a 2.2-fold increase in WT ACs cultures in the presence of IL-6 when compared with controls (*n*=3, *P*<0.001; Fig. 7h). Inhibition of STAT3 activation in CKO ACs abolished the increase in *Thbs1* mRNA (*n*=3, *P*>0.05), suggesting a tight link between STAT3 activation and *Thbs1* expression (Fig. 7h).

Next, we examined the TSP-1 protein content in non-activated and IL-6-activated astrocyte cultures. ACs were identified by GFAP/Aldh1L1 co-immunolabelling, as grey matter ACs may express no or low levels of GFAP^25^. IL-6 induced a significant rise (*P*=0.001) in the proportion of ACs with nSTAT3 labelling in WT cultures to 82.1±1.66% from 8.67±2.59% (*n*=3; Fig. 7i,j), whereas this was negligible or absent in all CKO ACs regardless the presence of IL-6 (*P*=0.521). In WT cultures, STAT3 activation resulted in significantly greater (*P*=0.025) proportion of ACs displaying TSP-1 immunoreactivity (42.42±3.4%) over non-treated cells (27.82±2.41%) or when compared with CKO cultures (*P*<0.001). In CKO ACs, there was no response to IL-6 (*n*=3, experiments/replicate cultures, *P*=0.292; Fig. 7i–k). These results indicate that TSP-1 protein expression in ACs is STAT3 driven.

Since TSP-1 can be secreted, we addressed whether it is released from ACs. Along with the increase of TSP-1 mRNA levels in activated ACs, immunoblotting and confocal imaging revealed that the intracellular TSP-1 content was reduced while TSP-1 was deposited on AC surfaces or in the extracellular matrix (Fig. 7l,m). In contrast, CKO ACs already had weaker TSP-1 bands, and this was not reduced upon activation to the extent seen for the WT cultures. We then measured the amount of TSP-1 released from ACs by enzyme-linked immunosorbent assay (ELISA). We observed significantly higher levels of TSP-1 concentration (2.08±0.10 pg ml^−1^) in the supernatant of IL-6-treated ACs in the WT group over the CKO cultures (1.53±0.09 pg ml^−1^) or controls (*n*=3, *P*<0.01; Fig. 7n). In summary, our experiments provide novel evidence that TSP-1 is re-expressed via STAT3 signalling, and it is also concomitantly released from the cells.

## Discussion

We provide definitive evidence that, despite the histological resemblance to ACs found in inhibitory glial scars in direct CNS injuries, remotely activated grey matter ACs promote structural plasticity and circuit integrity in the adult CNS. We describe novel mechanisms underlying this effect by showing that: (1) remote activation of ACs and their process formation in the vicinity of axotomized facial neuronal cell bodies is STAT3 dependent, despite the limited inflammatory response; (2) STAT3-dependent AC activation is required for the recovery of functional synaptic input onto surviving motor neurons, and not only for supporting neuronal preservation; (3) a direct regulatory link exists between astrocytic STAT3 activation and the re-expression/release of the synaptogenic molecule TSP-1; (4) TSP-1 re-expression, in part, is directly responsible for the remote AC-mediated recovery of excitatory input onto facial motor neurons in adult plasticity.

ACs responding to remote cues extend processes and tend to remain in their original domains without proliferation^6^,^19^. We provide both ultrastructural and immunohistochemical evidence that the formation of perineuronal astrocytic processes is mediated via STAT3 phosphorylation despite the lack of a significant inflammatory response. In our *in vivo* experiments, the majority of reactive ACs displayed nSTAT3 labelling, leading to a cascade of transcriptional events^26^. However, cytoplasmic pSTAT3 may also directly influence cell migration and process outgrowth, as found in fibroblasts, by binding to Rac1 activator β-PIX, modulating cytoskeletal re-organization^27^. Our findings show that STAT3 signalling is also a key element in remote reactive transformation of ACs, explaining the role of STAT3 activation seen in previous studies^18^,^28^. This could be potentially triggered by injured neurons via release of cytokines, such as IL-6 (refs 29, 30, 31).

What are the effects that remotely activated ACs exert through this pathway then? ACs with a reactive phenotype have often been regarded as cells inhibitory for repair^9^. However, recent evidence suggests that the outcome of glial reaction to CNS damage may vary depending on regional differences^32^,^33^ and on the severity of injury, and may contribute to tissue preservation in cases of moderate insults to neurons^34^,^35^. Our findings are in line with these observations. It is possible that the limited inflammatory cell response in the facial nucleus^36^ allows ACs to display a reactive but neuroprotective phenotype, indicating a default protective role of AC activation.

We provide evidence that astrocytic STAT3 signalling plays a direct and important role in supporting the integrity of the neuronal network, albeit they are likely to do so in conjunction with other cells. We demonstrate that ACs can independently support neuronal integrity via STAT3 signalling, using purified AC–neuron co-cultures. Indeed, microarray studies indicate that astrocytic STAT3 activation may induce neuronal survival by expression of molecules responsible for antioxidant defence^37^. This includes glutathione synthase, whose deficiency leads to less efficient protection against glutamate excitotoxicity^38^. Although, SCs can also support neuronal viability via c-Jun^39^, we cannot be entirely certain whether reduction in STAT3 activation in SCs could have contributed to an impaired trophic effect in CKO mice. The relevance of this possibility is yet to be addressed. Furthermore, it is plausible that ACs exert their protective effects in combination with microglia, too. Their interaction through purinergic gliotransmission has been shown to be protective^40^. We also found that the relative increase of the availability of reactive ACs to microglia corresponds with enhanced neuronal preservation. Other factors, such as limited lymphocyte influx, have shown to be less relevant in influencing survival in this model^36^.

Apart from protecting against the harmful environment, ACs can directly boost neuronal viability. Several reactive AC-derived trophic factors and cytokines have also been implicated in contributing to neuron survival^41^. There is also evidence for AC-induced long-term potentiation^42^,^43^,^44^,^45^, which may increase synaptic activity-induced neuronal survival^46^,^47^. This may occur when glutamatergic synapses are restored on neurons^48^,^49^,^50^,^51^,^52^. Whether ACs can also mediate survival by re-establishing balanced excitatory synaptic input needs to be examined in more detail.

Do perineuronal reactive ACs orchestrate structural plasticity to support circuit integrity? The involvement of reactive processes in reorganizing connections is suggested by descriptive studies showing enhanced glial process extension in areas with active synapse formation^17^,^19^,^53^,^54^. Initially, reactive microglial and perhaps AC processes may remove synapses from injured motor neurons during the first stages of synaptic rearrangements^55^,^56^. Our findings suggest the possibility that during the early phases of synapse stripping, microglial cells play a more significant role than do reactive ACs as the initial reduction in synapses was independent from the extent of early astrocytic response. However, the eventual structural recovery of synaptic input on motor neurons corresponded well with the relative availability of reactive ACs and their processes over microglial presence. Indeed, the numbers of activated microglia/macrophages were equal in the WT and CKO groups, yet synapse densities were reduced in the latter. These results suggest that reactive ACs promote the recovery of neuronal connections. Specifically, our electrophysiological findings and synapse density analysis indicate the restoration of functional afferents on motor neurons.

Thus, it was pertinent to ask whether AC activation can directly and independently facilitate excitatory synaptic rearrangements. AC-derived synaptogenic molecules were shown to stabilize excitatory synapses *in vitro*^14^ and in development^13^,^57^,^58^. Although the joint action of TSP-1 and TSP-2 is required for postnatal plasticity^58^, using TSP-1 knockout mice, we show that TSP-1 alone has a significant role in synaptic rearrangements in the adult brainstem. We therefore asked whether reactive ACs can supply TSP-1 to neurons in the recovery phase. We show that reactive ACs are the major populations that significantly upregulate TSP-1 expression proximal to the site of axotomy. The contribution of cells other than ACs in TSP-1-mediated adult plasticity is less likely, although in development, TSP-1 was seen in phagocytic cells and thrombospondin immunoreactivity could be also detected in microglia around dying neurons in adult mice^24^. Our study suggests that it is possibly a result of phagocytic internalization of neuronal debris or extracellular TSP-1 content, as *Thbs1* mRNA was almost exclusively detected in ACs and neurons. We propose that contribution by neurons to the increase of TSP-1 expression is modest. The number of *Thbs1* mRNA-labelled neurons was only marginally raised upon injury, and individual neurons did not seem to increase TSP-1 production. Indeed, in CKO mice in which neuronal STAT3 function is not affected, the increase in both TSP-1 mRNA and protein levels was almost abolished in tissue samples. These results indicate that the main source of TSP-1 is reactive ACs following axotomy, and our experiments conducted using purified AC cultures strengthen our conclusion. Using IL-6-stimulated ACs in culture, we demonstrated direct STAT3 binding to the TSP-1 promoter, increased mRNA levels and rapid loss of intracellular TSP-1 content concomitant with raised TSP-1 protein levels in supernatants upon astrocytic STAT3 activation. Our work provides direct evidence for STAT3-regulated TSP-1 expression and release.

Other mechanisms or factors may also be responsible for synaptic remodelling via activated ACs. Our study indicated a greater reduction in synapse recovery in CKO mice with impaired AC activation compared with that seen in TSP-1 KO mice. Recent evidence suggests that other AC-derived factors, such as glypican 4 and 6, may also play a role in neonatal plasticity^59^. *In vitro* PY2 purinergic signalling and type IV collagen/α1β1 integrin-mediated cascades also lead to an increase in TSP-1 expression^60^,^61^. We provide a novel regulatory mechanism for TSP-1 and show direct evidence for its involvement in reactive AC-mediated structural synaptic plasticity in adulthood.

Finally, what is the relevance to novel therapies in neurology? A common characteristic feature of CNS trauma or advanced neurodegenerative disease is widespread AC activation. It is essential to understand to what degree these astrocytic phenotypic changes represent pathology rather than an adaptive protective response. Emerging evidence suggests that the astrocytic effect may vary, even if the primary pathogenic trigger is derived from glia^62^. Microarray studies based on selective sampling of ACs in various neurodegenerative conditions have revealed both degeneration- and regeneration-related features^63^,^64^. In our reductionist paradigm of selective motor neuron injury, in which ACs are activated primarily by neuronal injury, reactivity leads to a restorative outcome. This approach provided an opportunity to dissect out the beneficial aspects of AC reaction that may also be relevant in disease. We present a novel regulatory mechanism mediated via astrocytic STAT3 signalling, which plays a key role in adult plasticity and network integrity in combination with other cells. Remotely activated ACs promote the recovery of excitatory input on surviving motor neurons by upregulation of TSP-1 expression. Further genomic and proteomic approaches are necessary to better understand the complexity of the AC response in neurodegeneration and trauma. This may open up opportunities to develop new neuroprotective strategies.

## Methods

### Animals and transgenic models

For modelling diminished AC activation, 8–10-week-old adult male GFAP-STAT3-CKO mice (CKO) were used (received from Prof. M. Sofroniew). Briefly, the transgenic mice were generated by crossing the *73.12 GFAP-Cre* mouse line^65^ with *STAT3-loxP* mice using C57BL/6 mice as background. The *loxP* sites flanked exon 22 of the STAT3 gene encoding a tyrosine residue (tyr705) that is critical for its activation, resulting in the inhibition of STAT3 activation specifically in GFAP-expressing cells in crossed CKO mice. These mice show comparable development and phenotypes of ACs to that seen for adult WT mice^12^. For WT control, 8–10 week-old adult male *GFAP-Cre*^−*/*−^*/STAT3-loxP* littermates and/or age-matched background C57BL/6 mice were used. For synapse recovery analysis, in addition to the above, 8–10-week-old adult male *Thbs1*^*tm1Hyn*^ mice (TSP-1 KO, C57BL/6J background) were also used. These mice are homozygous for *Thbs1* deletion (Jackson Labs).

### Surgical facial axotomy

All experimental procedures were carried out in accordance with the UK Scientific Procedures Act (1986) and guidelines set out by the International Association for the Study of Pain guidelines for the care and use of animals. The right facial nerve of 8–10-week-old male mice or postnatal P11–12 male mice was transected at its extracranial course near the stylomastoid foramen under fluothane (2%) anaesthesia with oxygen (1.5 l h^−1^). Before surgery, a subcutaneous injection of buprenorphine (Vetergesic; 0.1 mg kg^−1^) was administered to minimize pain and discomfort together with antibiotics (penicillin and streptomycin) to minimize potential infection. The adult animals were killed by a lethal injection of phenobarbital (300 mg kg^−1^) in humane conditions following brief anaesthesia by fluothane (2%) at different time points post axotomy (1, 5, 7, 14, 28 days).

### AC cultures

To generate ACs for both monocultures and neuronal co-cultures, cerebral cortices of P1 transgenic GFAP-STAT3-CKO mice, WT littermates, C57BL/6 WT mice (Harlan Olac & Charles River) and TSP-1 KO mice (Jackson Lab) were prepared as described in previous studies^66^. In general, this method establishes a purity of over 95% for ACs with less than 1% microglia. For our *in vitro* experiments, 98% pure AC cultures were used. In some cases, further purification steps were necessary to achieve this. To do so, immunopanning or complement-mediated cell lysis methods were applied to remove fibroblasts according to published protocols (Supplementary Methods). ACs were cultured for 6 weeks (mature ACs) in 10% fetal bovine serum, 1 mM GlutaMAX in Dulbecco’s modified Eagle medium (Life Technologies) to provide cells for *in vitro* assays. To evaluate the effects of AC reactivity, mature ACs were plated onto PDL coated coverslips (5 × 10^3^cells per well) in 24 well plates or in T25 flasks. After 2–3 days, the medium was replaced with Sato’s serum-free medium before treatment with IL-6 (50 ng ml^−1^), after which the cells were either immunostained or lysed for protein (complete lysis-M, EDTA-free buffer, Roche, UK) or RNA extraction. The supernatants were kept for analysing TSP-1 content by ELISA.

### AC–neuron co-culture assays

To produce purified cortical AC–neuron co-cultures in which ACs were replaced by their GFAP-STAT3-CKO or littermate counterparts, initially a mixed glial–neuron co-culture was produced using WT E18 embryos, similarly to previously published protocols^22^. To ablate contaminating and proliferating cell populations such as ACs, progenitor cells, fibroblasts and microglia, cytosine arabinoside (1 mM AraC, Sigma-Aldrich) was added to the cultures between days 5–7, while the medium was supplemented with AC-conditioned medium. This significantly reduced the number of ACs in the cultures (<6%). For the survival assays, mature ACs derived from either GFAP-STAT3-CKO mice or their WT littermates were re-added to the purified neuronal cultures. Cells were cultured for 21 days in total. See also Supplementary Methods.

### ChIP

To detect physical binding of STAT3 to the TSP-1 promoter region in ACs activated by IL-6, ChIP was performed using the Magna ChIP A/G Kit (Millipore) according to the manufacturer’s instructions. Briefly, 10^7^ mature ACs were fixed in 1% formaldehyde for 10 minutes followed by quenching with glycine for 5 min. ACs were washed in phosphate-buffered saline (PBS) before cell and nuclear lysis, which was performed in the presence of protease and phosphatase inhibitors. The isolated chromatin was sonicated to obtain DNA fragments of 200–1,000 base pairs. Then, immunoprecipitation was performed overnight at 4 °C, using a rabbit anti-STAT3 antibody (Cell Signalling, clone 79D7) bound to A/G magnetic beads. After isolation and elution, reverse cross-linking was performed according to standard protocols. DNA was then purified and analysed by PCR using a primer pair designed to detect a predicted STAT3 binding site^67^ along the TSP-1 promoter. The size of the PCR product was 176 bp. Forward primer: 5′-TGGCTTCCTCTGTGGTCTCT-3′. Reverse primer: 5′-GTCAAGGTCATGGGATGGTC-3′.

### Synthesis of digoxigenin-labelled RNA probes

To synthesize the probe for TSP-1 *in situ* hybridization, RNA from the brain of a P8 mouse was extracted using ISOLATE RNA Mini Kit (Bioline, UK), following the manufacturer’s instructions for RNA isolation. The isolated RNA was reverse transcribed into complementary DNA (cDNA) using the cDNA Synthesis Kit (Bioline) using a standard protocol for the Oligo dT primer. The resultant cDNA was amplified using standard PCR protocols. Murine TSP-1-specific primer sequences used were 5′-GTTCGTCGGAAGGATTGTTA-3′ for the forward primer and 5′-TCTATTCCAATGGCAACGAG-3′ for the reverse primer^68^. The size of the PCR product was 733 bp. Adaptor sequences were added to the original pair of primers to create attachment sites for the SP6 and T7 polymerases for the synthesis of RNA probes. cDNA from the first round of PCR was further amplified to produce the template for RNA probe synthesis, using the above forward primer with T7 phage promoter sequence (5′-TAATACGACTCACTATAGG-3′) added to its 5′ end and the above reverse primer with SP6 sequence (5′-ATTTAGGTGACACTATAGA-3′) added to its 5′ end. The SP6 and T7 promoter sequence allowed the antisense and sense RNA probe to be synthesized respectively. After confirming the identity of the PCR product by sequencing, digoxigenin (DIG)-labelled RNA probes were synthesized using the DIG RNA Labelling Kit (SP6/T7) (Roche) according to the manufacturer’s instructions. The yield of labelling (31.25 ng μl^−1^) was estimated using standardized dot blot protocol.

### *In situ* hybridization

TSP-1-specific *in situ* hybridization was performed following instructions described previously^69^, with minor modifications. In brief, frozen coronal mice brain sections (10 μm) were treated with proteinase K (200 ng ml^−1^ in PBS; Roche) for 20 s before fixing in 4% paraformaldehyde (PFA). After permeabilization, acetylation and prehybridization treatments, hybridization with prepared DIG-labelled probes (1:400 in hybridization buffer) was carried out at 60 °C overnight. Following stringency washing, the hybridized probe was recognized with alkaline phosphatase-conjugated DIG-specific antibody (1:5,000; Roche) for 1 h at 25 °C and the signal was then visualized with NBT/BCIP (Roche).

### Reverse transcription and qPCR

For measurements of *Thbs1* mRNA levels, mouse brainstems were snap-frozen and 300-μm thick coronal sections were cut, including the FN, using a cryostat (Leica). CL and IL FN were isolated using a purpose-made 0.8 mm punch needle; then, the tissue was triturated using a pestle. For *in vitro* samples, RNA was extracted from 5 × 10^5^ ACs.

Total RNA was isolated using the ISOLATE II RNA Mini Kit (Bioline) according to the manufacturer’s instructions. Reverse transcription was performed using 0.1–1 μg of total RNA, with the Tetro cDNA Synthesis Kit (Bioline) according to the defined protocol. qPCR was performed using SsoAdvanced Universal SYBR Green Supermix (Bio-Rad) in the Bio-Rad CFX96 thermocycler. Primers were chosen from the primePCR library (Bio-Rad) with a preference of intron-spanning primers when available. The *Thbs1* assay ID was qMmuCID0014108 and the *GAPDH* (glyceraldehyde 3-phosphate dehydrogenase) assay ID was qMmuCED0027497. Gene expression data were analysed using the relative quantification (*ddCt)* method using *GAPDH* as housekeeping gene to evaluate quantitative variation.

### Tissue processing

For immunohistochemistry, mice were perfused with 4% PFA solution in PBS under phenobarbital anaesthesia. Frozen blocks were cut at 10-μm thickness to gain a series of coronal brainstem sections, using a cryostat (Leica). For electron microscopy, after the same anaesthesia, mice were preperfused with 20 ml of 0.1 M HEPES buffer containing nitrates followed by perfusion with 6% formaldehyde in 0.1 M HEPES buffer at pH 7.4 containing 2 mmol l^−1^ calcium chloride 4 weeks post axotomy. The brains were kept in 0.1 M HEPES buffer for 1 h before trimming. Following infiltration by Lowicryl HM20 (Taab Laboratories) selected resin blocks were trimmed for ultrathin sectioning (Leica Ultracut UCT, Leica), stained with lead citrate/uranyl acetate, mounted on nickel grids and viewed by electron microscopy.

### Immunolabelling and imaging

For immunohistochemistry, following standard protocols, frozen sections were blocked in 10% normal goat serum (NGS) and permeabilized in 0.3% Triton X-100 (Sigma-Aldrich; in PBS) at RT for 30 min. They were then stained with primary antibodies in NGS (3%) and Triton X-100 (0.1% in PBS) at 4 °C overnight followed by species-specific secondary antibodies in PBS for 1 h and DAPI/Hoechst (100 ng ml^−1^) for 5–10 min at RT. For immunocytochemistry, on day 21, the coverslips were washed in PBS four times before fixing in 4% PFA (Sigma-Aldrich), then were blocked in 5% NGS in PBS. The cells were permeabilized in conjunction with the application of primary antibodies at RT for 45 min (2% NGS, 0.1% Triton X-100 in PBS, Sigma-Aldrich). Primary antibodies were used as follows: rabbit anti-Aldh1l1, 1:200 (Abcam, ab87117), goat anti-Arginase1, 1:100 (Abcam, ab60176), rabbit anti-activated caspase-3, 1:300 (Cell Signalling, P175), rat monoclonal anti-CD11b, 1:1,000 (Serotec, clone 5C6), rabbit anti-p-FAK, 1:1,000 (Life Technologies, 44624G), rabbit anti-GFAP, 1:500 (DAKO, Z0334), mouse monoclonal anti-GFAP, Cy3-conjugated, 1:500 (Sigma-Aldrich, clone FN-15), rabbit anti-IBA1, 1:500 (Wako, 019-19741), rat anti-Lamp2, 1:500 (Abcam, clone GL2A7), mouse anti-MAP-2, 1:100 (Sigma-Aldrich, clone HM-2), mouse monoclonal anti-NeuN, 1:200 (Millipore, clone A60), rabbit anti-PSD-95, 1:200 (Thermo Scientific, clone 6G6-1C9), mouse anti-S100, 1:500 (BD Biosciences, clone 19/S100B), rabbit monoclonal and mouse monoclonal anti-STAT3, 1:200 (Cell Signalling, clones 79D7 and 126H6, respectively), rabbit monoclonal anti-pSTAT3 (Tyr705), 1:100 (Cell Signalling, clone D3A7), mouse anti-SYT-1, 1:250 (Synaptic Systems, clone 41.1), mouse monoclonal anti-TSP-1, 1:50 (Santa Cruz, clone A6.1). This was followed by incubation with species-specific secondary antibodies for 30 min at RT. Images were taken either by light, fluorescent or confocal microscopes (Leica). The specificity for all antibodies were pretested by the companies. At first applications of primary antibodies, secondary antibodies alone were also used as negative controls. For analysis, either the Leica Application Suite software (Leica), Fiji or ImageJ v1.26 software was used.

### Western blotting and ELISA

Western blots were performed according to standard protocols (Life Technologies). Electrophoresis was run in 4–12% gradient NuPAGE Novex Bis-Tris Pre-Cast Gels (Life Technologies) and followed by protein transfer to a PDVF membrane (Life Technologies) according to standard protocols. Membranes were then washed three times in PBS 1 × with 0.2% Tween at pH 7.4. Blocking was performed in PBS 1 × , 0.2% Tween, 5% dry milk powder (Marvel). Membranes were stained with the primary and secondary antibodies diluted in the same blocking solution. Primary antibodies used were as follows: mouse monoclonal anti-β-actin, 1:20,000 (Abcam), mouse monoclonal anti-GFAP, 1:500 (Sigma-Aldrich), rabbit monoclonal anti-STAT3, 1:1,000 (Cell Signalling), rabbit monoclonal anti-pSTAT3 (Tyr705), 1:1,000 (Cell Signalling), mouse monoclonal anti-TSP-1, 1:200 (Santa Cruz). Detection was performed by exposing the membrane to the Amersham ECL Prime Western Blotting Detection Reagents (GE Healthcare). Chemiluminescence on membranes was detected and imaged with the Alliance 4.7 CCD Image System (UVITEC).

To detect TSP-1 release by ACs, a sensitive chemiluminescent ELISA was used according to standard protocols provided by Thermo Scientific. Briefly, the supernatants were ultracentrifuged first to concentrate the content of molecules above 50 kDa, using Amicon Ultra tubes (Millipore). Ninety-six well plates were coated with either standard dilutions of TSP-1 (0.5–20 pg ml^−1^ range) or with the concentrated samples (1:5 volume) in the coating buffer (0.2 M Na carbonate/bicarbonate, pH 9.4) at 4 °C overnight. TSP-1 dilution served as positive control and coating with buffer alone represented negative control. Non-specific binding sites were blocked in 2% BSA in the standard diluent (0.1 M phosphate, 0.15 M Na-chloride, 0.05% Tween 20, pH7.2; Sigma-Aldrich). For specific TSP-1 detection, a mouse TSP-1-specific antibody (1:1,000; Santa Cruz, clone A6.1) was applied, followed by a HRP-conjugated mouse-specific secondary antibody (1:50,000; Vector). The SuperSignal ELISA Femto chemiluminescent substrate (Thermo Scientific) was used for chemiluminescent detection, which was analysed by the GloMax plate-reader (Promega) at the wavelength of 420 nm. The antibodies were diluted in 1:5 strength of the standard diluent (50 μl total volume). The experiments included technical duplicates with biological triplicates.

### Image analysis for immunohistochemistry and western blots

In general at least 3–4 sections were taken for analysis 150 μm apart along the whole rostrocaudal axis of the FN. Cell counts, measurements of MAP-2 IR area fraction, intensity (optical density) of GFAP labelling and integrated density measurements for CD11b immunoreactivity were carried out in both axotomized and CL sides. LASAF (Leica) and ImageJ/Fiji applications were used to analyse areas of the entire FN in each tissue section according to stereotaxic parameters. The values were normalized to the non-axotomized CL side/background in the same section for accurate comparison between the groups. The methods for cell identification and counts for ACs, microglia, SCs and neurons have been described in the results. Western blot densitometry was performed by standard protocols using ImageJ/Fiji applications. Quantitative comparisons were performed based on normalization to density values of β-actin within the same blot.

### Analysis of AC processes and synapse density

We used classical ultrastructural criteria^21^ for quantifying AC processes and synapses. Astrocytic processes were identified by their irregular, concave shape, glycogen granules and intermediate filament bundles in a relatively electron-lucent cytoplasm. Electron microscopic images (86–112) were analysed per group, and the proportion of neuronal perimeter contacted by astrocytic endfeet was determined for 12–14 neurons. To examine STAT3-induced cytoskeletal changes, GFAP immunoreactivity was analysed *in vivo* in AC processes and F-actin was visualized by phalloidin in cultured ACs. In axotomized FN, AC processes showing GFAP immunoreactivity were counted 15 μm away from the soma^19^. For *in vitro* analysis, we counted ACs with strong cortical F-actin fibres, which were rounding up but kept connections with radial fibres extending to filopodia or processes. This represented the activated phenotype in contrast to resting cells that displayed flat morphology with mostly parallel filaments.

Axosomal and axodendritic synapses were identified by the presence of presynaptic vesicles and electron-dense postsynaptic densities. Synapse density was expressed as number of synapses per 1 micrometre length along the cell body or dendritic membrane. For immunohistochemistry-based synapse quantification, we followed a previously developed method^58^. Briefly, for quantification of synapse densities in the FN of mouse brains, four coronal sections per animal were immunostained for pre- and postsynaptic markers, synaptotagmin-1 and PSD-95, respectively. Then sections were scanned by confocal laser microscopy in 5 μm depth (optical section width 0.5 μm, 10 optical sections each). The parameters for scanning were set up for WT brain sections, and the same imaging parameters were used for transgenic animals. Merged single optical section images at 1 μm intervals were analysed using ImageJ (v1.0, NIH) for which the ImageJ puncta analyzer plug-in application was provided by Dr C. Eroglu^70^ (developed by B. Wark in Prof B. Barres’ laboratory). Threshold values were set to the CL side in each section. The number of colocalized pre- and postsynaptic puncta (five optical planes per section; 20 images per brain) was counted. Average synaptic density per imaged area (0.01 mm^2^) was calculated for each condition, and was normalized to the non-affected CL side.

### Electrophysiology

Whole-cell patch clamp recording of facial motor neurons was performed on coronal brainstem slices (225-μm thick) prepared from WT and CKO P21–24 old male mice 2 weeks following axotomy. Mice were killed by decapitation, in accordance with UK animal usage legislation. Tissue samples for genotyping were collected during tissue preparation. Synaptic currents were analysed and fitted using Electrophysiology Data Recorder V3.2.1 and Whole Cell Analysis Program V4.2.7 (Strathclyde Electrophysiology Software, Strathclyde University, UK), and were defined to occur if their amplitude was >1.5 times the s.d. of the current noise and their 10–90% decay time was longer than their rise time. Solutions used in electrophysiological studies were obtained from Sigma-Aldrich and described as follows. Slices were prepared in bicarbonate-buffered solution containing (mM) 126 NaCl, 24 NaHCO_3_, 1 NaH_2_PO_4_, 2.5 KCl, 1 MgCl_2_, 2 CaCl_2_, 1 Na-kynurenate to block glutamate receptors bubbled with 95% O2/5% CO2, pH 7.4. Electrodes contained internal solution comprising (mM) 130 Cs-gluconate, 4 NaCl, 0.5 CaCl_2_, 10 HEPES, 10 BAPTA, 4 MgATP, 0.5 Na_2_GTP, 2 K-Lucifer yellow, pH set to 7.3 with CsOH (*E*_Cl_=−88 mV). Pipette series resistance was 5–9 MΩ and electrode junction potentials were compensated. Slices were superfused at 24±1 °C with HEPES-buffered solution containing (mM) 144 NaCl, 2.5 KCl, 10 HEPES, 1 NaH_2_PO_4_, 2.5 CaCl_2_, 10 glucose, 0.1 glycine (to co-activate NMDA receptors), 0.005 strychnine (to block glycine receptors), pH set to 7.4 with NaOH, bubbled with 100% O_2_.

### Statistical analysis

All experiments included both biological and technical replicates, and were repeated three times at least. For *in vivo* experiments, the subjects were not randomized and two animals have been excluded from analysis due to an unsuccessful surgical procedure. The assessor was blinded by another investigator to the allocation of assays or animals during outcome assessment. The sample size was estimated from pilot experiments. Data and graphs are presented as mean±s.e.m, and ‘*n*’ values refer to the number of cells, cultures, tissue samples or animals analysed per group. GraphPad Prism 5 (GraphPad Software) was used to generate graphs and to perform tests for distribution and statistical significance. Data were analysed using two-tailed, unpaired *t*-test for comparison of two groups, which was referred to as ‘*t*-test’ in the text. One-way analysis of variance was applied for comparison of multiple groups and two-way analysis of variance was used to analyse independent variables in different groups. Bonferroni *post hoc* test was applied to analyse data pairs unless stated otherwise. Statistical significance was accepted at *P*-values of <0.05. *P, **P, ***P indicate <0.05, <0.01, <0.001, respectively. Non-significant *P*-values were labelled as ^NS^*P* in the text.

## 

## Author contributions

G.E.T. carried out part of the cell counting and analysis, western blots, qPCR, and performed the ChIP. S.S. conducted and analysed electrophysiological experiments guided by R.T.K. D.B. performed analysis on astrocyte and dendrite densities, and helped with the figures. K.L.A.-C. carried out the *in vitro* neuronal survival assay. N.K.L. and J.C.K. completed the *in situ* hybridization. C.Z. provided some of the transgenic mice and gave advice. R.J.M.F., R.T.K., J.W.F. provided feedback on the manuscript. A.L. developed the concept, directed the research, conducted and analysed experiments and wrote the manuscript.

## Additional information

**How to cite this article**: Tyzack, G. E. *et al*. Astrocyte response to motor neuron injury promotes structural synaptic plasticity via STAT3-regulated TSP-1 expression. *Nat. Commun.* 5:4294 doi: 10.1038/ncomms5294 (2014).

## Supplementary Material

Supplementary Information Supplementary Figures 1-4, Supplementary Methods and Supplementary References
