# Supplementary material for: Astrocyte response to motor neuron injury promotes structural synaptic plasticity via STAT3-regulated TSP-1 expression
Source: Nat Commun. 2014 Jul 11;5:4294. doi: 10.1038/ncomms5294 (PMC4104454; doi:10.1038/ncomms5294)
Supplement: Supplementary Information — Supplementary Figures 1-4, Supplementary Methods and Supplementary References [file ncomms5294-s1.pdf]

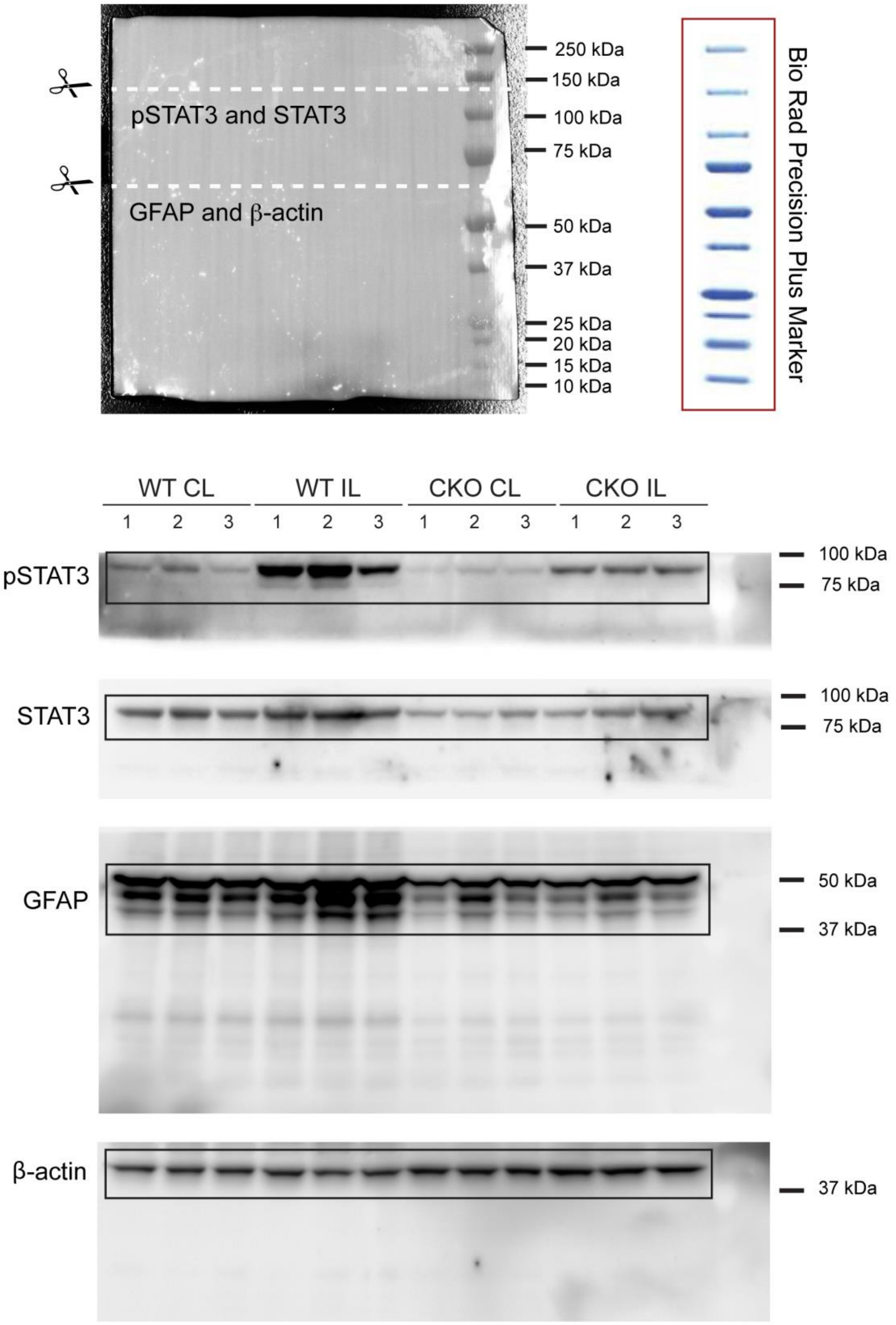

Supplementary Figure 1 | Whole blots for Figure 1n (WB). Blotted membranes were cut to enable simultaneous detection of multiple proteins in the same sample.

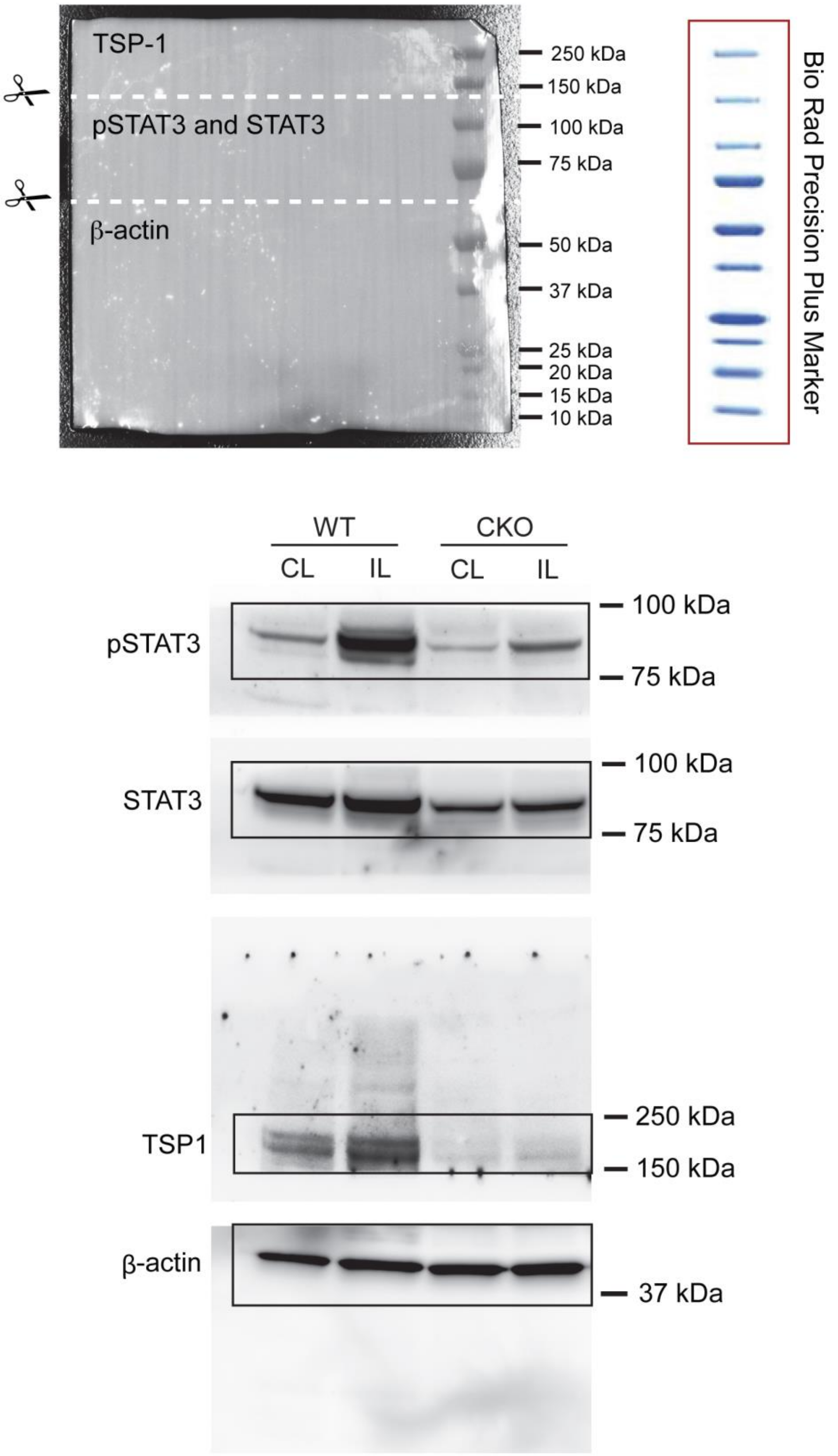

Supplementary Figure 2 | Whole blots for Figure 7e (WB). Blotted membranes were cut to enable simultaneous detection of multiple proteins in the same sample.

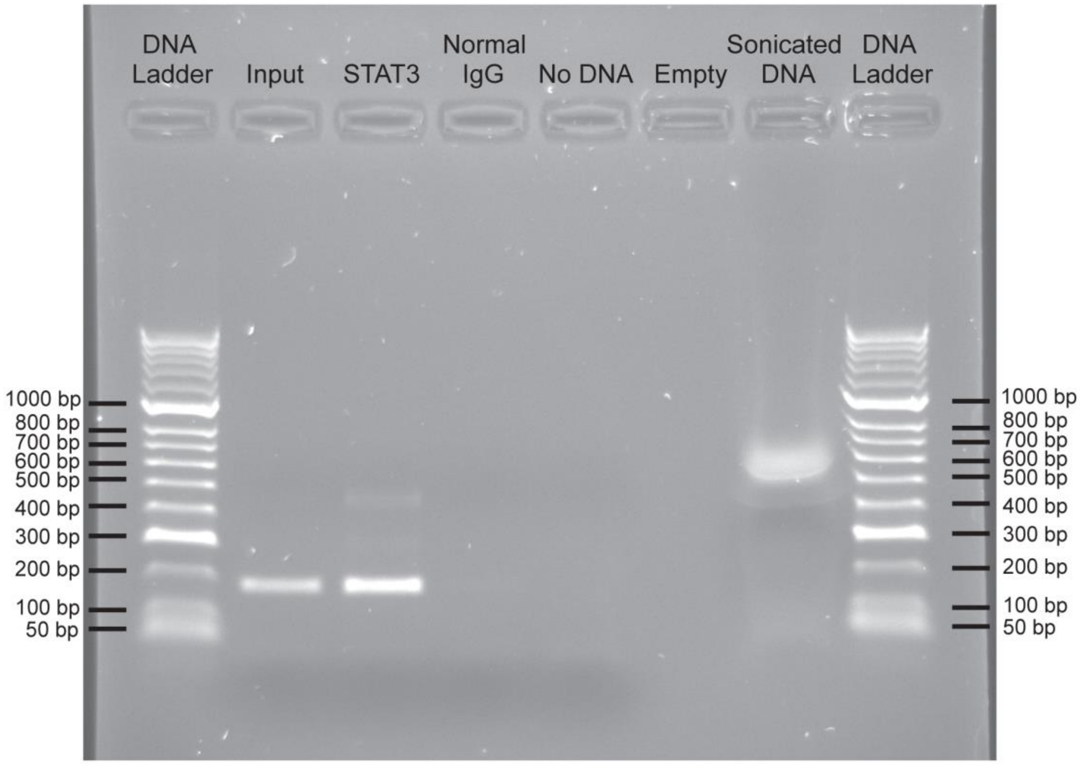

Supplementary Figure 3 | Whole gel for Figure 7g (ChIP).

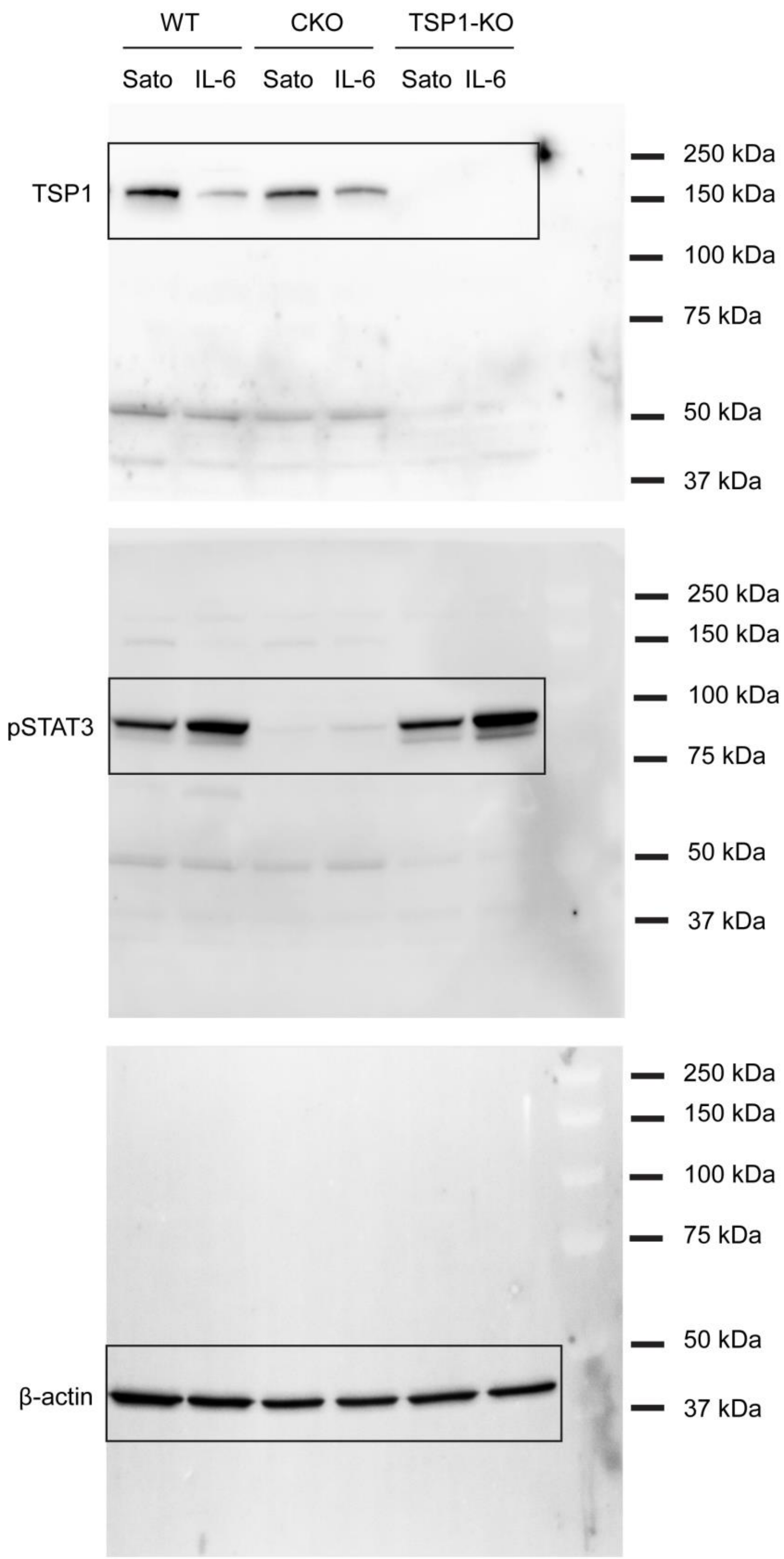

Supplementary Figure 4 | Whole blots for Figure 7m (WB). Blotted membranes were stripped to enable detection of multiple proteins in the same sample.

Supplementary Methods

Astrocyte cultures

Astrocytes were prepared by using previously described protocols as referenced in the main text. Cells were isolated from cerebral cortices of either 1-2 day old GFAP-STAT3-CKO or TSP-1 KO mouse pups and WT littermates in Mg^{2+}/Ca^{2+} free HBSS-0.1mM HEPES solution (Life Technologies). Following enzymatic dissociation (0.25% Trypsin (Sigma-Aldrich) in Mg^{2+}/Ca^{2+} free HBSS) the pellet was resuspended in 10ml of fresh DMEM-CM (DMEM, 10% fetal bovine serum, 1mM GlutaMAX™ (Life Technologies), penicillin-streptomycin-fungisone antibiotic complex, (1:100, Life Technologies). Cells were plated in poly-D-lysine (20 µg ml^{-1}) coated flasks at a density of 1-2 x 10^7 cells per 75 cm^2 surface area to allow expansion in 10%FBS-DMEM until day 7. Flasks were then shaken for 24 hours on a rotary platform at 300 rpm at 37°C to remove top dwelling microglia and progenitor cells. The supernatant containing the floating cells was discarded, the remaining cells washed thrice with 10ml Hanks Mg^{2+}/Ca^{2+} free solution. Then, astrocytes were cultured in fresh DMEM-CM. Between days 10 and 14, two 24 hour long pulses of cytosine arabinoside (1mM AraC, Sigma-Aldrich) treatment was applied and followed by washes in PBS two days apart to kill rapidly proliferating populations, such as progenitor cells, endothelial cells and meningeal cells/fibroblasts. Astrocytes were fed every 3 days by replacing half of their medium with fresh DMEM-CM. At weeks 3 and 5 in culture selective trypsinization was used to passage astrocytes at a high density of 3-5 x 10^6 cells per 25 cm^2 surface area, avoiding contamination by remaining fibroblasts and cultured at 5% CO_2 at 37°C C for 6 weeks in total. In general, this method provides a purity of over 95% for astrocytes with less than 1% microglia. For our *in vitro* experiments 98% pure astrocyte cultures were used. In some cases further purification steps were necessary to achieve this. To do so, immunopanning or complement-mediated cell lysis methods were applied using an anti-Thy1.1 antibody (1:1 supernatant, Serotec) according to previously published purification protocols[1–5] to remove the remaining fibroblast population. Purity of astrocyte cultures was verified by immunocytochemistry, using the following primary antibodies: rabbit anti-Aldh1l1, 1:200 (Abcam, ab87117) mouse monoclonal anti-GFAP, Cy3-conjugated, 1:500 (Sigma-Aldrich,

clone FN-15), rabbit anti-IBA1, 1:500 (Wako, 019-19741), mouse IgM anti-O4, 1:200 (Millipore, clone 81), mouse monoclonal anti-fibronectin, 1:500 (Sigma-Alrdrich, clone FN-15), anti-Thy1.1, 1:1 supernatant (Serotec) rabbit anti Von Willebrand Factor, 1:250 (Dako, #A0082).

Astrocyte-neuron co-cultures

To produce purified astrocyte-neuron (AC-N) co-cultures, initially a mixed glial-neuron co-culture was produced using wild type (WT) E18 embryos, according to previously published protocols as indicated in the main manuscript. Cortices of WT mice were dissected and processed for enzymatic dissociation (0.25% Trypsin (Sigma-Aldrich) in Mg^{2+}/Ca^{2+} free HBSS, Life Technologies). The cell suspension was triturated with polished Pasteur pipettes (3 sizes), then cells were plated in a medium containing high glucose [1.25%] DMEM, 10% fetal calf serum, 2mM Glutamax (Life Technologies) at 10^5 cell/coverslip density (500µl medium per well/24 well plate) on poly-D-lysine pre-coated coverslips (100 µg ml^{-1} for 24 hours, Sigma-Aldrich). Following a 3-4 hour incubation period at 37˚C (5% CO_2), the medium was replaced by 1ml of neuron culture medium (NCM: Neurobasal, 1:50 B27, 2mM Glutamax, Life Technologies). This medium allows neuronal maturation but reduces the proportion of contaminating cells by limiting their survival and proliferation potential. To ablate contaminating and proliferating cell populations such as astrocytes, progenitor cells, fibroblasts and microglia, cytosine arabinoside (1mM AraC, Sigma-Aldrich) was added to the cultures between days 5-7 for 48-72 hours, while the medium was supplemented with astrocyte conditioned medium (1:1). This significantly reduced the number of astrocytes in the cultures (<6%). For the survival assays, mature astrocytes derived from either GFAP-STAT3-CKO mice or their WT littermates were re-added to the purified neuronal cultures. Cultures were then fed every 7 days by replacing half of the existing medium (500µl) with fresh NCM. Cells were cultured for 21 days in total.

Supplementary References

1. Brockes, J. P., Fields, K. L. & Raff, M. C. Studies on cultured rat Schwann cells. I. Establishment of purified populations from cultures of peripheral nerve. *Brain Res.* **165**, 105–118 (1979).

2. Lakatos, A., Franklin, R. J. M. & Barnett, S. C. Olfactory ensheathing cells and Schwann cells differ in their in vitro interactions with astrocytes. *Glia* **32**, 214–225 (2000).

3. Plant, G. W. *et al.* Purified adult ensheathing glia fail to myelinate axons under culture conditions that enable Schwann cells to form myelin. *J. Neurosci.* **22**, 6083–91 (2002).

4. Lakatos, A., Smith, P. M., Barnett, S. C. & Franklin, R. J. M. Meningeal cells enhance limited CNS remyelination by transplanted olfactory ensheathing cells. *Brain* 598–609 (2003).

5. Foo, L. C. *et al.* Development of a method for the purification and culture of rodent astrocytes. *Neuron* **71**, 799–811 (2011).
